# Standardizing the estimation of ischemic regions can harmonize CT perfusion stroke imaging

**DOI:** 10.1007/s00330-023-10035-1

**Published:** 2023-08-12

**Authors:** Daan Peerlings, Edwin Bennink, Jan W. Dankbaar, Birgitta K. Velthuis, Bart J. Emmer, Jan W. Hoving, Charles B. L. M. Majoie, Henk A. Marquering, Henk van Voorst, Hugo W. A. M. de Jong, On behalf of the CLEOPATRA (cost-effectiveness of CT perfusion for patients with acute ischemic stroke) healthcare evaluation and the DUST (Dutch acute stroke) study investigators

**Affiliations:** 1 Department of Radiology, University Medical Center Utrecht, Utrecht, 3584CX The Netherlands; 2 Image Sciences Institute, University Medical Center Utrecht, Utrecht, 3584CX The Netherlands; 3 Department of Radiology and Nuclear Medicine, Amsterdam University Medical Centers, Location Academic Medical Center, Amsterdam, 1105AZ The Netherlands; 4 Department of Biomedical Engineering and Physics, Location Academic Medical Center, Amsterdam University Medical Centers, Amsterdam, 1105AZ The Netherlands

**Keywords:** Brain, Ischemic stroke, Four-dimensional computed tomography, Clinical protocols, Perfusion imaging

## Abstract

**Objectives:**

We aimed to evaluate the real-world variation in CT perfusion (CTP) imaging protocols among stroke centers and to explore the potential for standardizing vendor software to harmonize CTP images.

**Methods:**

Stroke centers participating in a nationwide multicenter healthcare evaluation were requested to share their CTP scan and processing protocol. The impact of these protocols on CTP imaging was assessed by analyzing data from an anthropomorphic phantom with center-specific vendor software with default settings from one of three vendors (A–C): IntelliSpace Portal, syngoVIA, and Vitrea. Additionally, standardized infarct maps were obtained using a logistic model.

**Results:**

Eighteen scan protocols were studied, all varying in acquisition settings. Of these protocols, seven, eight, and three were analyzed with center-specific vendor software A, B, and C respectively. The perfusion maps were visually dissimilar between the vendor software but were relatively unaffected by the acquisition settings. The median error [interquartile range] of the infarct core volumes (mL) estimated by the vendor software was − 2.5 [6.5] (A)/ − 18.2 [1.2] (B)/ − 8.0 [1.4] (C) when compared to the ground truth of the phantom (where a positive error indicates overestimation). Taken together, the median error [interquartile range] of the infarct core volumes (mL) was − 8.2 [14.6] before standardization and − 3.1 [2.5] after standardization.

**Conclusions:**

CTP imaging protocols varied substantially across different stroke centers, with the perfusion software being the primary source of differences in CTP images. Standardizing the estimation of ischemic regions harmonized these CTP images to a degree.

**Clinical relevance statement:**

The center that a stroke patient is admitted to can influence the patient’s diagnosis extensively. Standardizing vendor software for CT perfusion imaging can improve the consistency and accuracy of results, enabling a more reliable diagnosis and treatment decision.

**Key Points:**

*• CT perfusion imaging is widely used for stroke evaluation, but variation in the acquisition and processing protocols between centers could cause varying patient diagnoses.*

*• Variation in CT perfusion imaging mainly arises from differences in vendor software rather than acquisition settings, but these differences can be reconciled by standardizing the estimation of ischemic regions.*

*• Standardizing the estimation of ischemic regions can improve CT perfusion imaging for stroke evaluation by facilitating reliable evaluations independent of the admission center.*

**Supplementary information:**

The online version contains supplementary material available at 10.1007/s00330-023-10035-1.

## Introduction

Clinical stroke research increasingly relies on multicenter CT perfusion (CTP) imaging [1, 2]. Yet, multicenter CTP imaging is afflicted by a substantial variation in the imaging protocols used across different centers [3]. This variation raises important questions about the consistency of scientific results and the validity of clinical guidelines.

The scan protocol and perfusion software can influence CTP results in numerous ways. Several acquisition settings, such as the tube voltage, exposure, and timing of the frames, have been assessed over the years and have resulted in a multitude of considerations [4–7]. The same holds for different preprocessing steps, such as determining the arterial input function or reducing noise, implemented by the perfusion software [8–10]. Moreover, perfusion algorithms and infarct estimations have been shown to characterize ischemia differently from each other [11–14]. In daily clinical practice, stroke patients are thus evaluated in various ways according to the protocols of their admission center.

To address the daily reality of stroke imaging, this paper presents the first study of real-world variation in CTP imaging protocols among stroke centers. For a large stroke healthcare evaluation, we assess the impact of scan protocols on CTP imaging by analyzing data from an anthropomorphic phantom with center-specific vendor software. Additionally, we explore the potential for standardizing vendor software to harmonize CTP images.

## Methods

### Phantom data for scan protocols

Stroke centers participating in the CLEOPATRA (cost-effectiveness of CTP for patients with acute ischemic stroke) healthcare evaluation were requested to share their scan protocol [15]. The CLEOPATRA healthcare evaluation combines data from multiple prospective endovascular thrombectomy trials in the Collaboration for New Treatments of Acute Stroke (CONTRAST) consortium [16–18]. In total, 1164 patients were eligible for CLEOPATRA: 228 from the MR CLEAN-NO IV trial, 120 from the MR CLEAN-MED trial, 251 from the MR CLEAN-LATE trial, 419 from the MR CLEAN Registry, and 146 from a local cohort.

The tube voltage (kVp), the exposure (mAs), and the timing of the frames from the CLEOPATRA stroke centers were input to an anthropomorphic digital phantom designed for a realistic CTP simulation of acute ischemic stroke that is entirely digital [19]. These parameters could be implemented in the phantom easily while giving a proper overview of the differences between centers.

The phantom combined MR brain images with CT imaging parameters. The (nondynamic) MR imaging of a healthy volunteer provided the brain parenchyma and the cerebral vascular system in high resolution (0.34 mm × 0.34 mm × 0.3 mm). On the MR brain images, we manually drew a ground truth infarct core (i.e., irreversibly damaged tissue) of 30 mL and a ground truth penumbra (i.e., salvageable tissue) of 55 mL in the right hemisphere, totaling to 85 mL of hypoperfused tissue.

The CT volumes that were produced from these MR images were of size 512 × 512 × 8 voxels (for each frame) with a voxel size of 0.5 mm × 0.5 mm × 5 mm. We added realistic CT noise to these CT volumes. The noise images were randomly generated with a standard deviation that corresponded to the noise in scans of a physical skull phantom made for a range of CT imaging parameters. (At 500 mAs, the standard deviation of white noise would be 3.7 HU for the digital phantoms used in this study. The actual noise images were adjusted to the reported mAs and were made spatially dependent with a kernel derived from the scans of the physical skull phantom.) For each scan protocol, ten noise realizations of the phantom were generated to take the effect of noise on CTP images into account.

The phantom could not generate noise for a tube voltage of 70 kVp because no scan data of the physical skull phantom was available for 70 kVp. Hence, for acquisitions at 70 kVp, the input parameters for the phantom were adjusted to 80 kVp while halving the mAs, conforming to the rule of thumb that an increase of 15% in tube voltage corresponds to a 50% decrease in tube current for the dose to stay the same [20].

### Perfusion analysis by vendor software

**Fig. 1:**
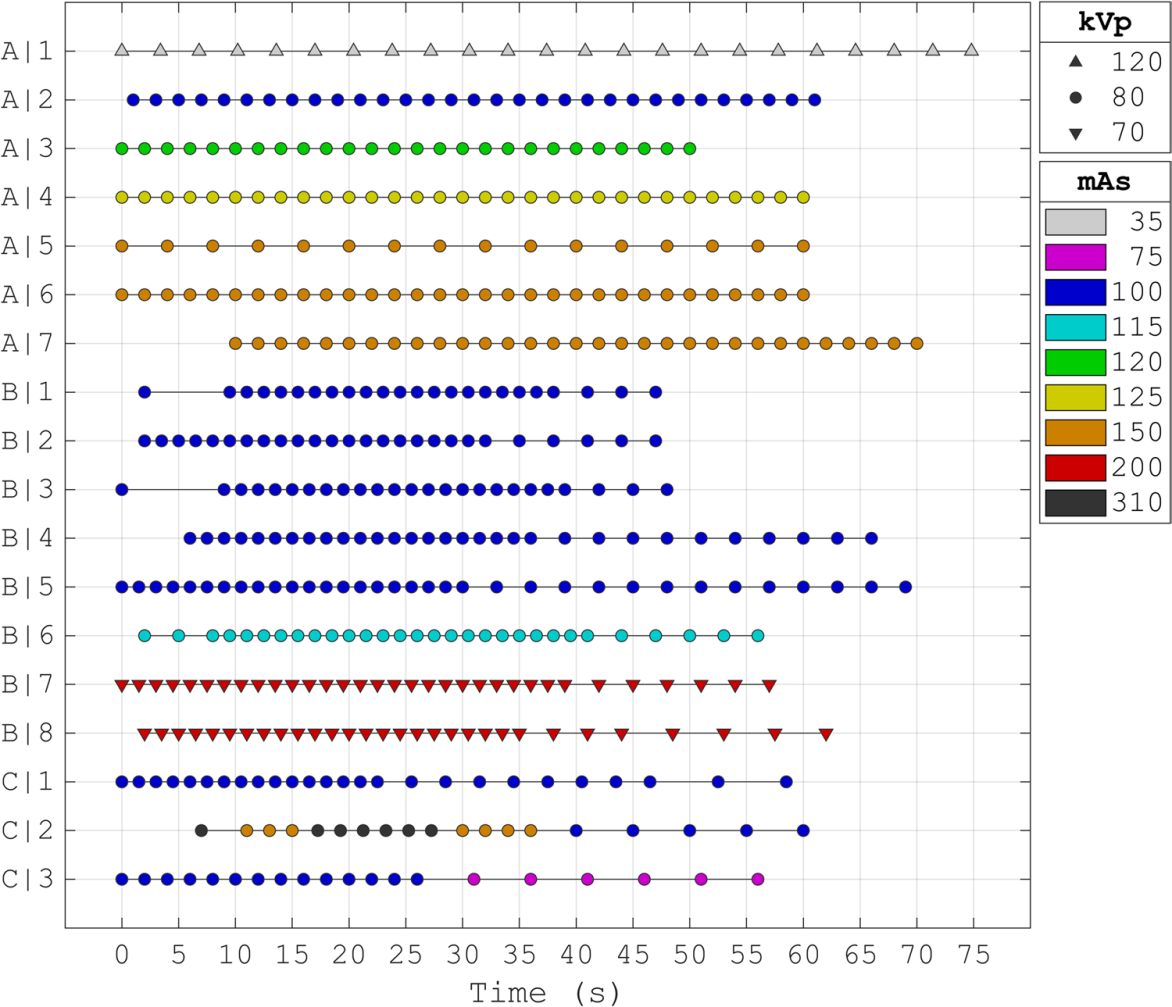
The scan protocols that were all shared upon request. Time is zero at the start of the contrast injection. Each of the eighteen scan protocols is denoted by a letter (A–C) indicating the vendor software and followed by a number, specifying the scan protocol

For each scan protocol (Fig. 1), the ten noise realizations of the phantom were analyzed with center-specific software from one of three vendors (A–C): CT Brain Perfusion (arrival-time-sensitive algorithm) from IntelliSpace Portal version 10.1 (Philips Healthcare), CT Neuro Perfusion from syngoVIA version VB40A-HF02 (Siemens Healthineers), and CT Brain Perfusion 2D (Bayesian algorithm) from Vitrea version 7.14 (Vital Images). For each analysis, we adhered to the default software settings and let the arterial input function be determined automatically. All further data processing and analysis were carried out with MATLAB (MATLAB, R2019b: The Mathworks Inc.).

The three vendor software did not all produce the same set of perfusion parameters. Vendor software A yielded a perfusion map of the cerebral blood flow (CBF), the cerebral blood volume (CBV), the mean transit time (MTT), and the time to peak (TTP). Vendor software B and C generated a time to maximum (TMAX) parameter map instead of a TTP parameter map. The TTP parameter is the time from the start of the scan until maximum enhancement. Loosely speaking, the TMAX parameter is the TTP parameter corrected for the arrival time of the arterial input function. In this paper, we sometimes write “TMAX or TTP” by which we mean TTP for vendor software A and TMAX for vendor software B and C.

The three vendor software did not all export the perfusion maps in the same way. The perfusion maps from vendor software A and B were exported as DICOM files that contained the actual parameter values. The parameter values from vendor software A were exported as integers whereas the parameter values from vendor software B were not rounded. The perfusion maps from vendor software C were exported as DICOM files that contained grayscale values (i.e., intensities ranging from 0 to 255). These grayscale values were rescaled to obtain the parameter values. The range with which the grayscale DICOM files were exported was 0–150 mL/100 g/min for the CBF, 0–10 mL/100 g for the CBV, 0–20 s for the MTT, and 0–15 s for the TMAX. For vendor software A, the exported images were upsampled from 256 × 256 voxels to 512 × 512 voxels (i.e., the original size of the phantom) by repeating each voxel 2 × 2 times.

### Ischemic stroke regions estimated by vendor software

The ischemic stroke regions were estimated from the perfusion maps by the vendor software with the default thresholds (Table 1). The volumes of the estimated ischemic stroke regions reported by the vendor software were logged. The segmentations of the estimated ischemic stroke regions could not be exported as DICOM files so we made screenshots of these segmentations instead (for illustrative purposes and to archive the results visually).

**Table 1:** The default thresholds to estimate the ischemic stroke regions for each vendor software. CBF is cerebral blood flow, CBV is cerebral blood volume, MTT is mean transit time, and TMAX is time to maximum. Values relative to the opposite hemisphere are indicated by an “r”

Software	Infarct core	Hypoperfused region	Perfusion algorithm
A	CBV < 2.0 mL/100 g and rMTT > 150%	rMTT > 150%	Singular value decomposition
B	CBV < 1.2 mL/100 g	CBF < 27.0 mL/100 g/min	Singular value decomposition
C	rCBV < 38% and TMAX > 2.30 s	TMAX > 2.30 s	Bayesian

### Ischemic stroke regions estimated by standardized method

From the perfusion maps that were generated by the vendor software, we estimated the ischemic stroke regions with a standardized method. Our aim was to provide a flexible framework to summarize the perfusion maps into different (ischemic stroke) regions. The model used for this standardization should be robust and generalizable. Little but clear training data should help to provide certainty to evident cases, leaving less certain cases to the predictive ability of the model.

We opted for a logistic model that was multivariable (i.e., more than one input variables) and multivariate (i.e., more than two output variates) [22]. In a multivendor context and from a theoretical perspective, it is preferable to include multiple perfusion parameters because it allows a fairer comparison between perfusion software. Since CTP differentiates between multiple ischemic stroke regions, it is natural to implement multiple outcomes for the tissue fate of a voxel in a single model. So, the logistic model we used to estimate the ischemic stroke regions reads:

$${\mathrm{P}}_{\mathrm{CORE}}={10}^{{\mathrm{S}}_{\mathrm{CORE}}}/\left(1 + {10}^{{\mathrm{S}}_{\mathrm{CORE}}}+ {10}^{{\mathrm{S}}_{\mathrm{PENUMBRA}}}\right),$$

$${\mathrm{P}}_{\mathrm{PENUMBRA}}={10}^{{\mathrm{S}}_{\mathrm{PENUMBRA}}}/\left(1 + {10}^{{\mathrm{S}}_{\mathrm{CORE}}}+ {10}^{{\mathrm{S}}_{\mathrm{PENUMBRA}}}\right),$$

$${\mathrm{P}}_{\mathrm{HEALTHY}}=1-{\mathrm{P}}_{\mathrm{CORE}}-{\mathrm{P}}_{\mathrm{PENUMBRA}},$$

Where

$${\mathrm{S}}_{\mathrm{CORE}}={\mathrm{C}}_{\mathrm{INT}}^{\mathrm{CORE}}+{\mathrm{C}}_{\mathrm{CBF}}^{\mathrm{CORE}}\times \mathrm{CBF}+{\mathrm{C}}_{\mathrm{CBV}}^{\mathrm{CORE}}\times \mathrm{CBV}+{\mathrm{C}}_{\mathrm{MTT}}^{\mathrm{CORE}}\times \mathrm{MTT}+{\mathrm{C}}_{\mathrm{TMAX}}^{\mathrm{CORE}}\times \mathrm{TMAX},$$

And

$${\mathrm{S}}_{\mathrm{PENUMBRA}}={\mathrm{C}}_{\mathrm{INT}}^{\mathrm{PENUMBRA}}+{\mathrm{C}}_{\mathrm{CBF}}^{\mathrm{PENUMBRA}}\times \mathrm{CBF}+{\mathrm{C}}_{\mathrm{CBV}}^{\mathrm{PENUMBRA}}\times \mathrm{CBV}+{\mathrm{C}}_{\mathrm{MTT}}^{\mathrm{PENUMBRA}}\times \mathrm{MTT}+{\mathrm{C}}_{\mathrm{TMAX}}^{\mathrm{PENUMBRA}}\times \mathrm{TMAX }.$$

The CBF is in mL/100 g/min, the CBV in mL/100 g, the MTT in seconds, and the TMAX in seconds. For each vendor software, the regression coefficients C followed from a logistic regression by maximum likelihood estimation. For vendor software A, the algorithm was changed to arrival-time-insensitive (yielding a TMAX parameter map) because variable scanning starting times would otherwise result in a TTP that is not suited as a predicting variable. The arrival-time-insensitive algorithm has no recommended threshold values to estimate the ischemic stroke regions so was otherwise not used in the comparison between vendor software.

To estimate the regression coefficients of the logistic model, training data were obtained from five patient CTP scans included in the DUST (Dutch acute stroke) study [23]. These scans were selected because of an infarct core and a penumbra that were easy to distinguish on the perfusion maps generated by an in-house developed model-based nonlinear regression method [24]. To obtain the ground truth classifications, we drew two regions of ten by ten voxels in what we considered to be 100% infarct core, 100% penumbra, and 100% healthy tissue for each of the five patient scans. Hence, the model was trained on the perfusion parameters of 1000 (= 2 × 10 × 10 × 5) voxels annotated as infarct core, 1000 voxels annotated as penumbra, and 1000 voxels annotated as healthy tissue. We obtained the perfusion maps for the training data by analyzing the patient scans with each vendor software in the same way as the phantoms.

The logistic models were applied to the exported perfusion maps of the phantoms, producing fuzzy segmentations of the ischemic stroke regions. We determined the volumes of the estimated ischemic stroke regions by adding the probabilities in the fuzzy segmentation [14].

### Assessment of CTP imaging

We assessed the impact of the scan protocol and the vendor software on both the perfusion parameters and the estimated ischemic stroke regions. For the perfusion parameters, we pooled the ten noise realizations for each scan protocol and depicted the values of the perfusion parameters within the infarct core, the penumbra, healthy white matter, and healthy gray matter with boxplots (given the ground truth regions in the phantom). For the estimated ischemic stroke regions, we depicted the volumes from the scan protocols with boxplots and reported the median, first quartile, and third quartile error of the volumes estimated by the vendor software and after standardization.

## Results

### Scan protocols and vendor software

All eighteen (A|1-C|3) scan protocols from the CLEOPATRA stroke centers were shared upon request (Fig. 1). Seven of the protocols were analyzed with vendor software A, eight with vendor software B, and three with vendor software C. The scan protocols varied considerably between centers in the exposure and timing of the frames.

For scan protocols at 80 kVp, the average exposure was between 100 and 150 mAs, except for scan protocols C|2 and C|3 with an average exposure of 196 mAs and 93 mAs respectively. For lower tube voltages (of 70 kVp in scan protocols B|7 and B|8), the average exposure was 200 mAs. For higher tube voltages (of 120 kVp in scan protocol A|1), the average exposure was 35 mAs.

Scan protocols A|1 and A|5 had a longer interval between frames during contrast enhancement (which was between 10 and 35 s): 3.4 s and 4.0 s for scan protocols A|1 and A|5 respectively compared to at most 2.0 s for the other scan protocols. Scan protocol A|7 had a delayed scanning starting time of 10.0 s. Also, scan protocol C|2 had only one frame well before contrast arrival (which was around 10 s).

### Examples of CTP imaging

**Fig. 2:**
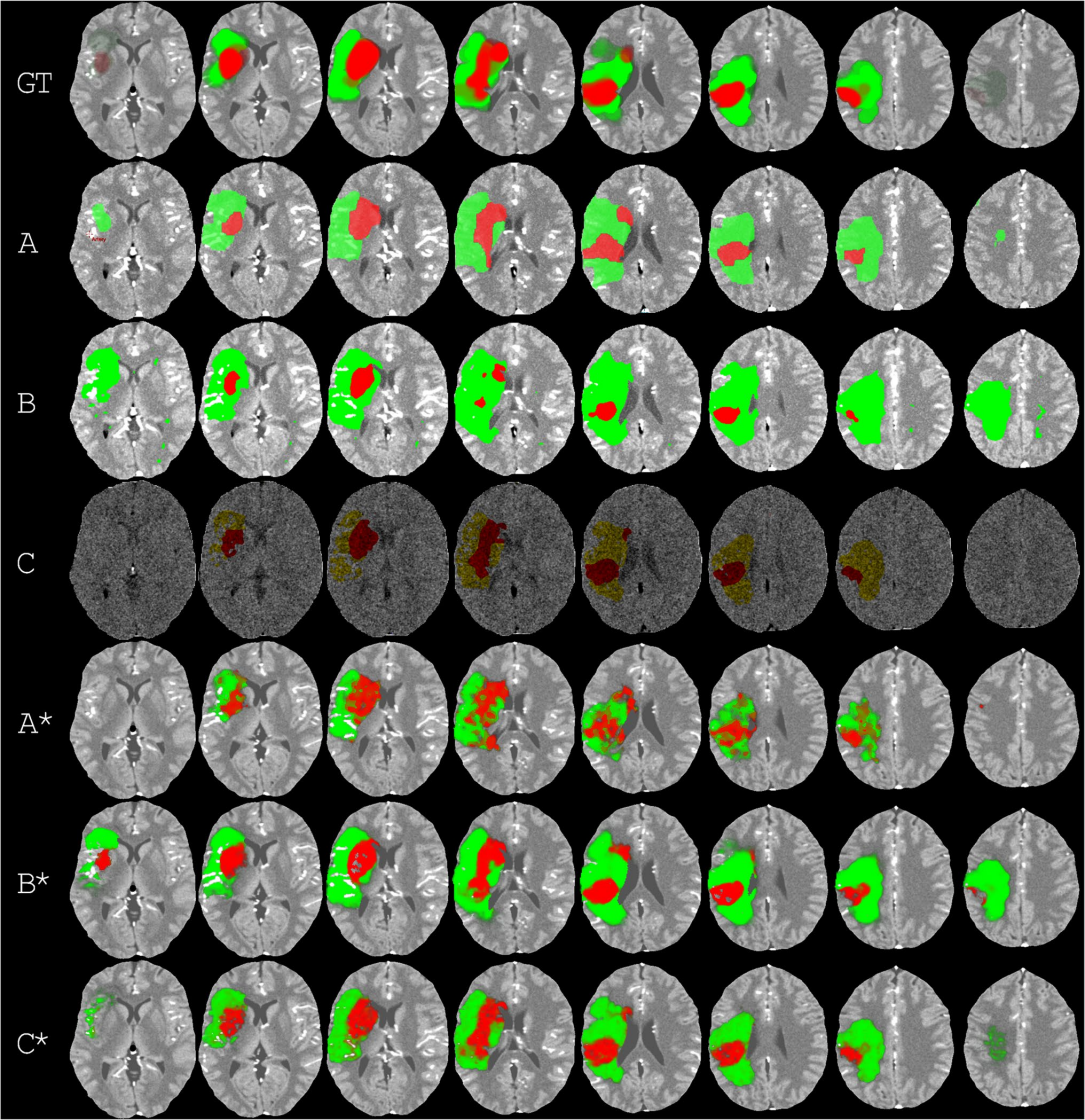
Examples of the ischemic stroke regions estimated by the vendor software and by the standardized method. The infarct core is in red and the penumbra is in green or yellow. Ideally, each column should be the same in all its rows. On the top row, the ground truth segmentations are shown. These segmentations are fuzzy because they were made on thinner MR slices. On the second to fourth row, the ischemic stroke regions estimated by the vendor software (**A**–**C**) are shown, obtained from screenshots. The screenshots from vendor software C are darker and noisier because they show the first frame instead of a maximum intensity projection. On the fifth to seventh row, the ischemic stroke regions estimated by the standardized method (A*–C*) are shown. The examples are the first noise realization from the representative scan protocols A|4, B|6, and C|1 (see Fig. 1)

**Fig. 3:**
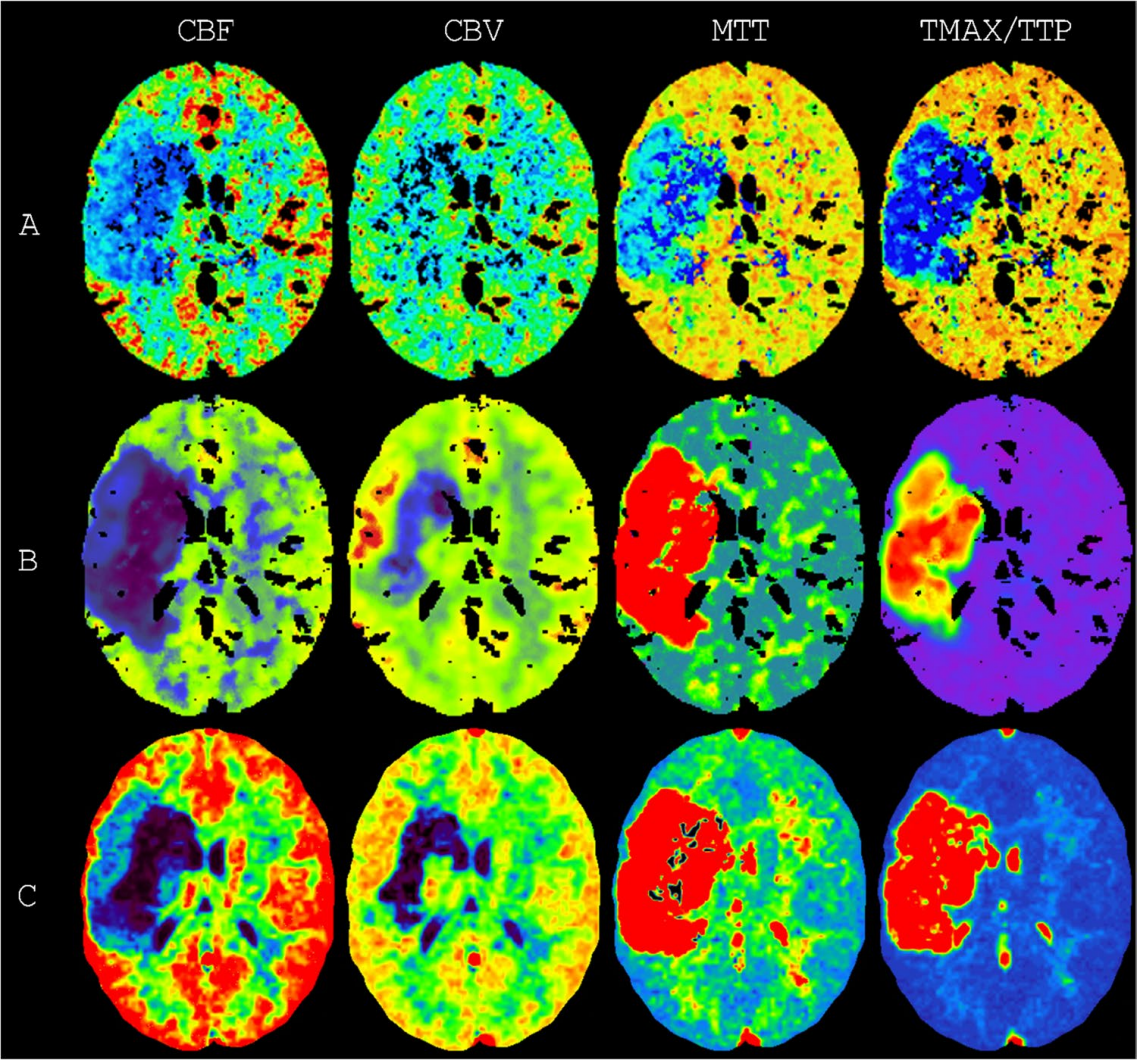
Examples of the perfusion maps generated by the vendor software. In each row, the perfusion maps from a different vendor software (**A**–**C**) are shown for a single slice of the phantom. In each column, a different perfusion map is shown for each vendor software. Ideally, each column should be the same in all its rows. CBF is cerebral blood flow, CBV is cerebral blood volume, MTT is mean transit time, TMAX is time to maximum, and TTP is time to peak. The color schemes were left unadjusted. The examples are the first noise realization from the representative scan protocols A|4, B**|**6, and C|1 (see Fig. 1)

**Fig. 4:**
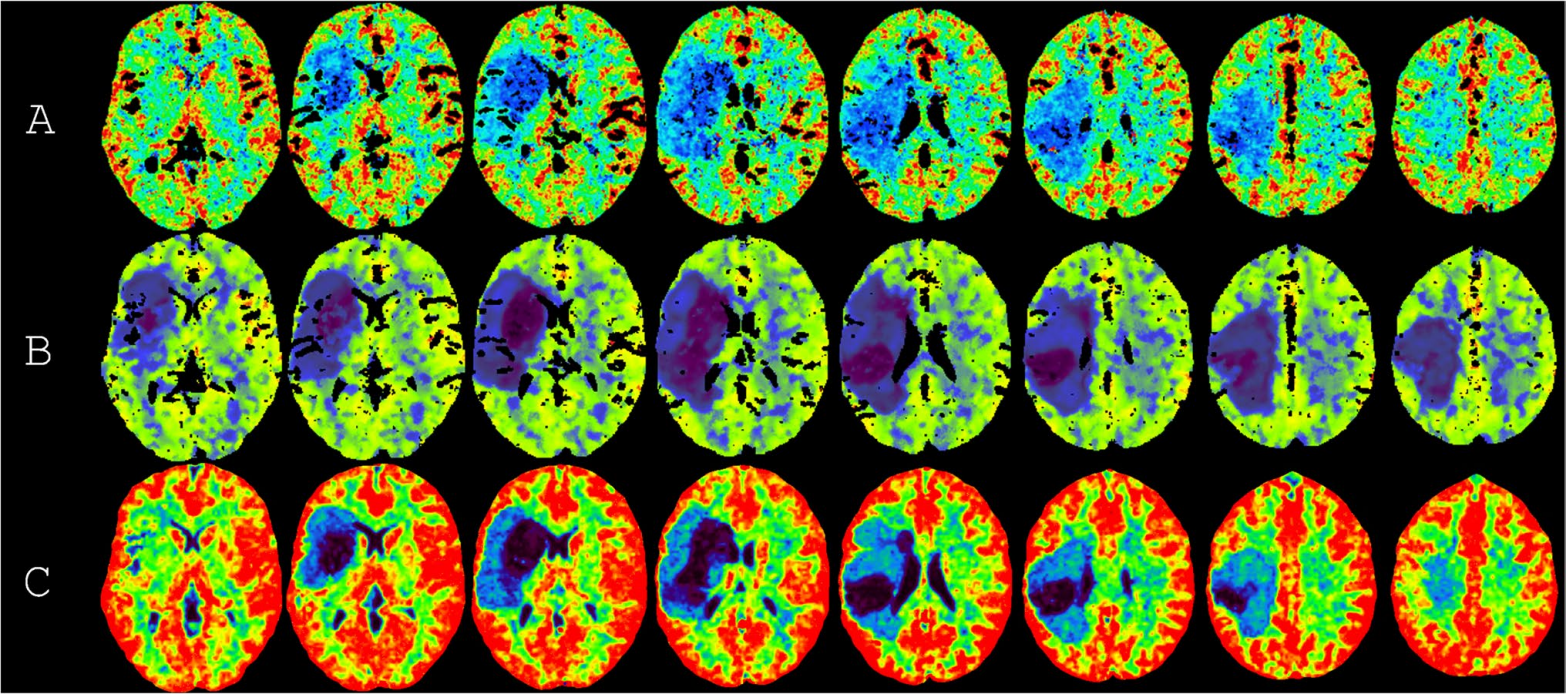
Examples of the cerebral blood flow parameter map generated by the vendor software. In each row, the cerebral blood flow from a different vendor software (**A**–**C**) is shown for all slices of the phantom. Ideally, each column should be the same in all its rows. The color schemes were left unadjusted. The examples are the first noise realization from the representative scan protocols A|4, B|6, and C|1 (see Fig. 1)

Figure 2 shows examples of the ischemic stroke regions estimated by the vendor software and after standardization (see Table 2 for the logistic regression coefficients for each vendor software). These estimated ischemic stroke regions were derived from the perfusion maps, shown in Fig. 3 for one of the eight slices. Additionally, the CBF parameter map is shown for all eight slices in Fig. 4. All eight slices for the CBV, MTT, and TMAX/TTP parameter maps can be found in the Supplementary Material. Between vendor software, both the ischemic stroke regions estimated by the vendor software (Fig. 2) and the perfusion maps (Fig. 3 and Fig. 4) were visually dissimilar.

**Table 2:** The logistic regression coefficients for each vendor software. *The coefficient for the cerebral blood flow (C*_*CBF*_*) is in* (mL/100 g/min)^−1^*, the coefficient for the cerebral blood volume (C*_*CBV*_*) is in (mL/100 g)*^*−1*^*, the coefficient for the mean transit time (C*_*MTT*_*) is in (seconds)*^*−1*^*, and the coefficient for either the time to maximum or the time to peak (C*_*TMAX*_*) is in (seconds)*^*−1*^

Software	Ischemic region	C_INT_	C_CBF_	C_CBV_	C_MTT_	C_TMAX_
A	Core	− 5.0	− 0.0391	− 4.74	− 0.39	2.8
	Penumbra	− 18.5	− 0.0313	0.73	− 0.56	3.2
B	Core	20.7	− 0.7049	− 8.00	− 0.29	2.9
	Penumbra	− 13.7	− 0.0045	0.54	0.33	2.9
C	Core	− 0.2	− 0.7486	4.70	− 0.41	1.7
	Penumbra	− 16.3	− 0.1747	3.87	0.48	1.7

It appears from Fig. 2 that the estimated ischemic stroke regions were harmonized, to a degree, after standardization. For vendor software B, the estimated penumbra in a slice resembled the ground truth penumbra in that slice and its adjacent slices. In particular, this seemed to result in a reduced estimation of the infarct core by vendor software B as well as an estimated hypoperfused region in the outer slices, where barely any hypoperfusion should exist. Hypoperfusion in the outer slices is also clearly visible on the perfusion maps generated by vendor software B (Fig. 4). These results may have been due to the filter size of vendor software B, which was 10 mm, i.e., twice the slice thickness. Vendor software A appears to generate the noisiest perfusion parameters.

### Assessment of CTP imaging

**Fig. 5:**
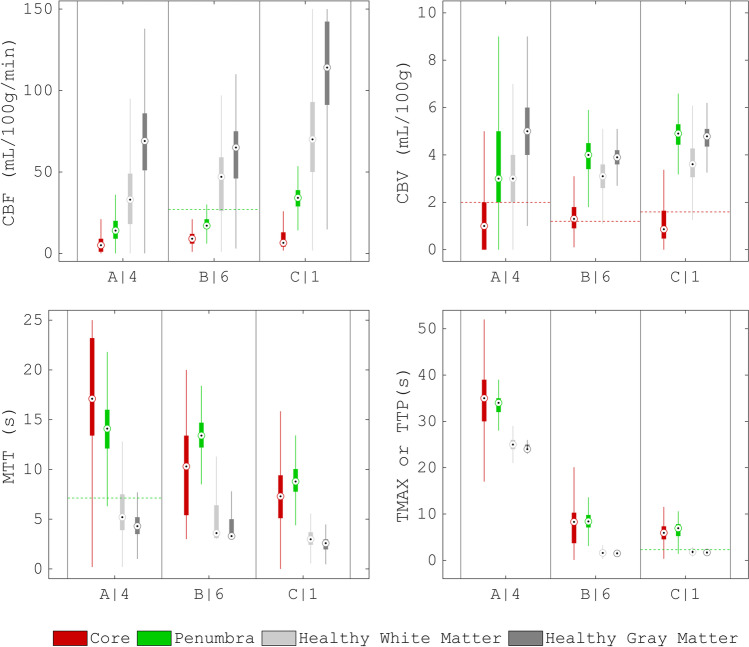
Boxplots of the perfusion parameters estimated by the vendor software. We pooled the ten noise realizations of the phantom for each scan protocol and show the results for scan protocol A|4, B|6, and C|1. The dashed horizontal colored lines indicate the thresholds given in Table 1, for which relative values were calculated as relative to the median value of the perfusion parameter in healthy matter. CBF is cerebral blood flow, CBV is cerebral blood volume, MTT is mean transit time, TMAX is time to maximum, and TTP is time to peak

Figure 5 shows boxplots of the perfusion parameters for one (representative) scan protocol per vendor software. We refer to the Supplementary Material for a similar overview of all the scan protocols.

**Fig. 6:**
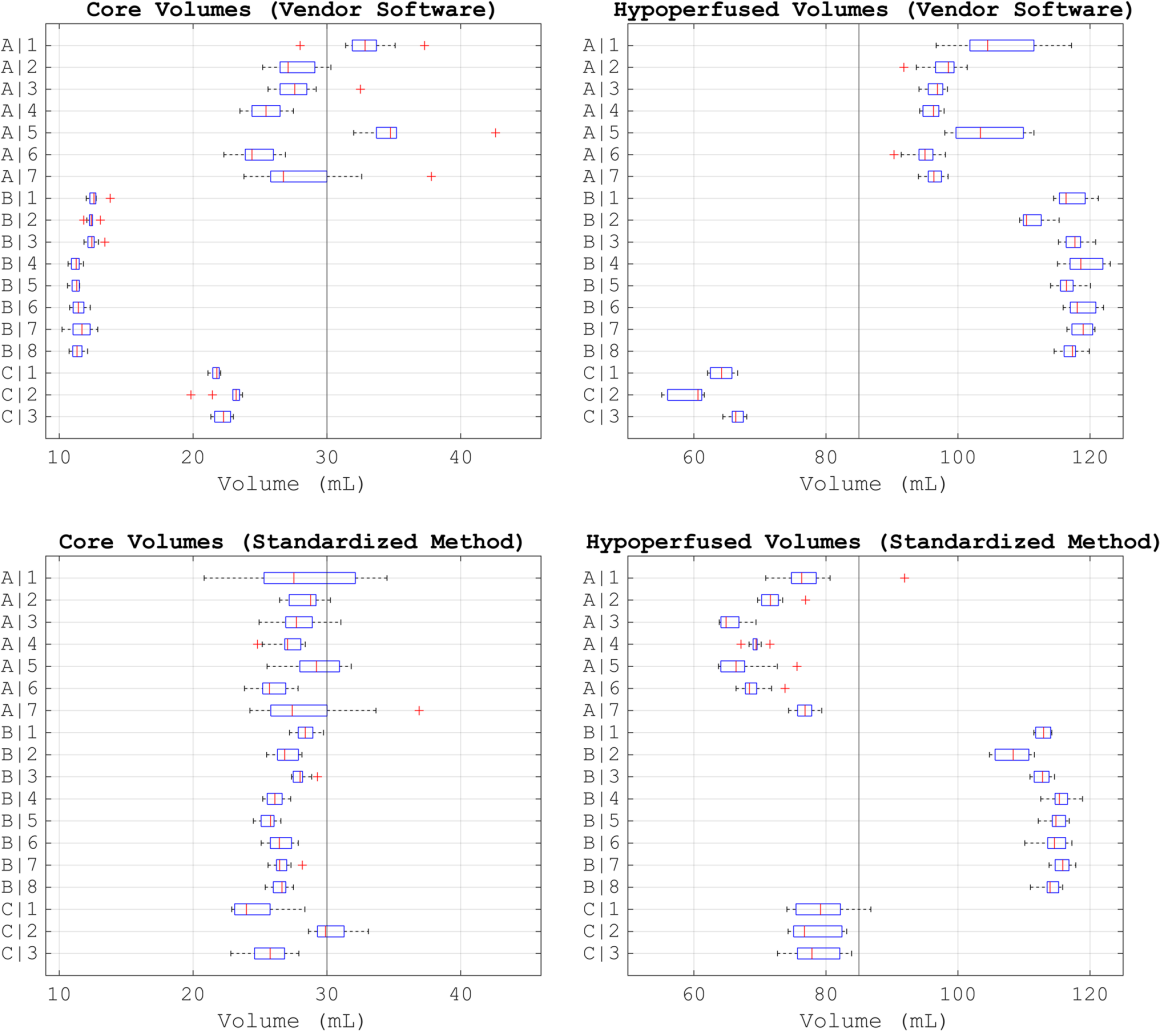
Boxplots of the volumes of the ischemic stroke regions estimated by the vendor software and by the standardized method. Eighteen scan protocols (A|1-C|3) were analyzed with center-specific software from one of three vendors (A–C). The vertical black lines indicate the ground truth volumes

Figure 6 shows the boxplots of the volumes of the estimated ischemic stroke regions for each scan protocol (additional boxplots can be found in the Supplementary Material). The median, first quartile, and third quartile error of the volumes estimated by the vendor software and by the standardized method are given in Table 3.

**Table 3:** Median [first quartile, third quartile] error of the volumes of the ischemic stroke regions estimated by the vendor software and by the standardized method. We pooled all noise realizations of the phantom. A positive error indicates overestimation

Ischemic region	Vendor software	Standardized method
Core (mL)	− 8.2 [− 18.1, − 3.5]	− 3.1 [− 4.2, − 1.7]
Hypoperfused (mL)	18.4 [10.2, 32.0]	− 3.2 [− 12.5, 28.8]

Figure 6 demonstrates that the differences between centers were mainly due to the vendor software. Vendor software A estimated the largest infarct cores and was the most sensitive to different noise realizations of the phantom. Vendor software B and C were both much less sensitive to the different noise realizations. Vendor software B estimated smaller infarct cores than vendor software C. Hence, three groups of estimated volumes according to vendor software clearly emerged.

Albeit much less than the vendor software, the scan acquisition protocol impacted the estimated volumes in some cases (Fig. 6). Scan protocols A|1 and A|5, with a longer interval between frames during contrast enhancement, resulted in volumes that deviated the most. Too few frames before contrast arrival may have increased the variance in the estimated infarct core for scan protocols A|7 and C|2 because of an increased noise in the CBV [25].

The estimated volumes of the infarct core were aligned between vendor software and scan protocols after standardization (Fig. 6 and Table 3). The estimated volumes of the hypoperfused region were still segregated, mainly between vendor software B and vendor software A and C (Fig. 6). The standardized method overestimated the hypoperfused region for vendor software B, which could be expected from the perfusion maps and which resulted in a wider interquartile range for the hypoperfused volumes (Fig. 4 and Table 3).

## Discussion

Our study evaluated CTP stroke imaging in a real-world setting and found that the estimated ischemia varied greatly between centers. The primary source of this variation was the perfusion software rather than the acquisition protocol. Previous research has already shown for patient data that vendor software can cause large differences in estimated ischemia and our study supports these findings with homogeneous phantom data representative of clinical variation [11, 26–28]. The homogeneous nature of our data, combined with the disparate outcome, suggests that multicenter CTP data and prevailing clinical guidelines may in fact hold limited validity. Hence, patients are likely evaluated variously at present, with both scientific and clinical consequences, depending on the software used to analyze their CTP scans.

Much of the variation between vendor software was due to the estimation of ischemia. While the perfusion maps were both qualitatively and quantitatively dissimilar, the standardized method resulted in a harmonized estimation of ischemia. This feasibility of harmonization implies that the perfusion parameters from the different vendor software actually contain a comparable level of information and can be equally valuable on the whole when properly assessed. We opted for a logistic model to standardize vendor software because of its ability to combine multiple perfusion parameters when characterizing ischemia, while being insusceptible to multicollinearity in its predictions, so that each vendor software could be assessed fairly based on all of their perfusion data [21]. Additionally, a logistic model is relatively easy to implement by vendors. Although similar models have been proposed in the past, they have not been applied in the context of harmonization [13, 14, 29–31]. Some variation in the estimated ischemia remained, demonstrating a desirable sensitivity to the acquisition and processing protocol. As a clear example, the overestimated hypoperfusion from vendor software B rightly resulted in divergent volumes. Thus, variability in CTP imaging resulted mainly from the vendor software but could be compensated for when estimating ischemia.

The acquisition protocol may require minimal guidelines to ensure consistent CTP imaging. Previous studies have already shown that acquisition settings can affect CTP images separately [4–7]. By examining existent acquisition protocols integrally, our findings suggest that the timing of the frames is the most consequential aspect of present scan protocols. Minimal requirements on this timing, such as a maximum interval during contrast enhancement and a minimum number of frames before contrast arrival, may be sufficient to level the variation that was due to the scan protocol. Hence, only little standardization of the acquisition protocol seems necessary to secure a harmonized CTP outcome when the same perfusion software is used.

Our study has some limitations. It is based on phantom data, which may not directly translate to patient data. Emulating anthropomorphic perfusion data and generating realistic scanner noise are both challenging tasks. Additionally, scanner-specific features such as the reconstruction algorithm are difficult to incorporate in a digital phantom. Besides, we did not consider the contrast medium injection protocol, which has been shown to affect CTP imaging as well and which may combine with aspects of the scan protocol [32]. For instance, shorter injection times may require shorter frame intervals to not overpass the contrast enhancement phase. Finally, an identical treatment of each vendor software was hampered by their different implementations, for example in the set, the size, and the value of the exported DICOM images, limiting the veracity of the standardization of the vendor software.

## Conclusion

We evaluated CTP imaging in a real-world setting and found that ischemia was estimated disparately between centers. The perfusion software, rather than the acquisition protocol, was the main cause of this variation. Still, the variation in estimated ischemia could be reconciled by incorporating all available perfusion data in a consistent way. Accordingly, we advocate for the harmonization of CT perfusion imaging by standardizing the estimation of ischemia.

### Supplementary Information

Below is the link to the electronic supplementary material.

Supplementary file1 (PDF 902 KB)

## Abbreviations

CBF: Cerebral blood flow
CBV: Cerebral blood volume
CLEOPATRA: Cost-effectiveness of CT perfusion for patients with acute ischemic stroke (healthcare evaluation)
CONTRAST: Collaboration for New Treatments of Acute Stroke (consortium)
CTP: Computed tomography perfusion
DUST: Dutch acute stroke (study)
MTT: Mean transit time
TMAX: Time to maximum
TTP: Time to peak

## Declarations

### Guarantor

The scientific guarantor of this publication is H.W.A.M. De Jong.

### Statistics and biometry

No complex statistical methods were necessary for this paper.

### Study subjects or cohorts overlap

Some study subjects or cohorts have been previously reported in

van Seeters, T., Biessels, G. J., van der Schaaf, I. C., Dankbaar, J. W., Horsch, A. D., Luitse, M. J., … & Velthuis, B. K. (2014). Prediction of outcome in patients with suspected acute ischaemic stroke with CT perfusion and CT angiography: the Dutch acute stroke trial (DUST) study protocol. *BMC neurology*, *14*(1), 1–8.

Van Der Hoeven, E. J., Dankbaar, J. W., Algra, A., Vos, J. A., Niesten, J. M., Van Seeters, T., … & Velthuis, B. K. (2015). Additional diagnostic value of computed tomography perfusion for detection of acute ischemic stroke in the posterior circulation. *Stroke*, *46*(4), 1113–1115.

Hamming, A. M., van Walderveen, M. A., Mulder, I. A., van der Schaaf, I. C., Kappelle, L. J., Velthuis, B. K., … & Algra, A. (2019). Circle of Willis variations in migraine patients with ischemic stroke. *Brain and behavior*, *9*(3), e01223.

van Seeters, T., Biessels, G. J., Kappelle, L. J., Van Der Schaaf, I. C., Dankbaar, J. W., Horsch, A. D., … & Schonewille, W. J. (2015). The prognostic value of CT angiography and CT perfusion in acute ischemic stroke. *Cerebrovascular diseases*, *40*(5–6), 258–269.

van Seeters, T., Biessels, G. J., Kappelle, L. J., van der Schaaf, I. C., Dankbaar, J. W., Horsch, A. D., … & Schonewille, W. J. (2016). CT angiography and CT perfusion improve prediction of infarct volume in patients with anterior circulation stroke. *Neuroradiology*, *58*(4), 327–337.

Horsch, A. D., Bennink, E., van Seeters, T., Kappelle, L. J., van der Graaf, Y., Mali, W. P., … & DUST Investigators. (2018). Computed tomography perfusion derived blood–brain barrier permeability does not yet improve prediction of hemorrhagic transformation. *Cerebrovascular Diseases*, *45*(1–2), 26–32.

### Methodology

• retrospective

• observational

• multicenter study

## References

[1] Nogueira RG, Jadhav AP, Haussen DC (2018). Thrombectomy 6 to 24 hours after stroke with a mismatch between deficit and infarct. N Engl J Med.

[2] Albers GW, Marks MP, Kemp S (2018). Thrombectomy for stroke at 6 to 16 hours with selection by perfusion imaging. N Engl J Med.

[3] Shankar JJS (2021). Variation in CT perfusion protocol has implications on defining irreversibly damaged ischemic brain parenchyma. Eur Radiol.

[4] Wintermark M, Maeder P, Verdun FR et al (2000) Using 80 kVp versus 120 kVp in perfusion CT measurement of regional cerebral blood flow. AJNR Am J Neuroradiol 21:1881–1884 PMC7974284 11110541

[5] Murphy A, So A, Lee TY (2014). Low dose CT perfusion in acute ischemic stroke. Neuroradiology.

[6] Manniesing R, Oei MTH, Van Ginneken B, Prokop M (2016). Quantitative dose dependency analysis of whole-brain CT perfusion imaging. Radiology.

[7] van Ommen F, Kauw F, Bennink E (2019). Effect of prolonged acquisition intervals for CT-perfusion analysis methods in patients with ischemic stroke. Med Phys.

[8] Fahmi F, Marquering HA, Borst J (2014). 3D movement correction of CT brain perfusion image data of patients with acute ischemic stroke. Neuroradiology.

[9] Ferreira RM, Lev MH, Goldmakher GV et al (2010) Arterial input function placement for accurate CT perfusion map construction in acute stroke. AJR Am J Roentgenol 194:1330–1336. 10.2214/AJR.09.2845 10.2214/AJR.09.2845 PMC3744327 20410422

[10] Mendrik AM, Vonken EJ, Van Ginneken B (2011). TIPS bilateral noise reduction in 4D CT perfusion scans produces high-quality cerebral blood flow maps. Phys Med Biol.

[11] Kudo K, Sasaki M, Yamada K (2010). Differences in CT perfusion maps generated by different commercial software: quantitative analysis by using identical source data of acute stroke patients. Radiology.

[12] Konstas AA, Lev MH (2010). CT perfusion imaging of acute stroke: the need for arrival time, delay insensitive, and standardized postprocessing algorithms?. Radiology.

[13] Flottmann F, Broocks G, Faizy TD (2017). CT-perfusion stroke imaging: a threshold free probabilistic approach to predict infarct volume compared to traditional ischemic thresholds. Sci Rep.

[14] Peerlings D, van Ommen F, Bennink E, et al (2022) Probability maps classify ischemic stroke regions more accurately than CT perfusion summary maps. Eur Radiol 32:6367–6375. 10.1007/s00330-022-08700-y 10.1007/s00330-022-08700-y PMC9381605 35357536

[15] Koopman MS, Hoving JW, van Voorst H (2022). Cost-effectiveness of CT perfusion for patients with acute ischemic stroke (CLEOPATRA)-Study protocol for a healthcare evaluation study. Eur Stroke J.

[16] Chalos V, Van De Graaf RA, Roozenbeek B (2020). Multicenter randomized clinical trial of endovascular treatment for acute ischemic stroke. The effect of periprocedural medication: acetylsalicylic acid, unfractionated heparin, both, or neither (MR CLEAN-MED). Rationale and study design. Trials.

[17] Treurniet KM, LeCouffe NE, Kappelhof M (2021). MR CLEAN-NO IV: intravenous treatment followed by endovascular treatment versus direct endovascular treatment for acute ischemic stroke caused by a proximal intracranial occlusion—study protocol for a randomized clinical trial. Trials.

[18] Pirson FAV (Anne), Hinsenveld WH, Goldhoorn R-JB et al (2021) MR CLEAN Late study protocol. Trials 15:1–13 10.1186/s13063-021-05092-0 PMC7903604 33627168

[19] Riordan AJ, Prokop M, Viergever MA (2011). Validation of CT brain perfusion methods using a realistic dynamic head phantom. Med Phys.

[20] Lira D, Padole A, Kalra MK, Singh S (2015). Tube potential and CT radiation dose optimization. AJR Am J Roentgenol.

[21] Farrar DE, Glauber RR (1967). Multicollinearity in regression analysis : the problem revisited. Rev Econ Stat.

[22] Ebrahimi Kalan M, Jebai R, Zarafshan E, Bursac Z (2021). Distinction between two statistical terms: multivariable and multivariate logistic regression. Nicotine Tob Res.

[23] van Seeters T, Biessels GJ, van der Schaaf IC (2014). Prediction of outcome in patients with suspected acute ischaemic stroke with CT perfusion and CT angiography: the Dutch acute stroke trial (DUST) study protocol. BMC Neurol.

[24] Bennink E, Oosterbroek J, Kudo K (2016). Fast nonlinear regression method for CT brain perfusion analysis. J Med Imaging.

[25] Li K, Chen G-H (2016) Noise characteristics of CT perfusion imaging: how does noise propagate from source images to final perfusion maps? Proc SPIE Int Soc Opt Eng 9783:978310. 10.1117/12.2216293 10.1117/12.2216293 PMC5710833 29200593

[26] Zussman BM, Boghosian G, Gorniak RJ et al (2011) The relative effect of vendor variability in CT perfusion results: a method comparison study. AJR Am J Roentgenol 197:468–473. 10.2214/AJR.10.6058 10.2214/AJR.10.6058 21785096

[27] Koopman MS, Berkhemer OA, Geuskens RREG (2019). Comparison of three commonly used CT perfusion software packages in patients with acute ischemic stroke. J Neurointerv Surg.

[28] Fahmi F, Marquering HA, Streekstra GJ et al (2012) Differences in CT perfusion summary maps for patients with acute ischemic stroke generated by 2 software packages. AJNR Am J Neuroradiol 33:2074–2080. 10.3174/ajnr.A3110 10.3174/ajnr.A3110 PMC7965595 22555577

[29] Kemmling A, Flottmann F, Forkert ND (2015). Multivariate dynamic prediction of ischemic infarction and tissue salvage as a function of time and degree of recanalization. J Cereb Blood Flow Metab.

[30] Goyal M, Ospel JM, Menon B et al (2020) Challenging the ischemic core concept in acute ischemic stroke imaging. Stroke 3147–3155. 10.1161/STROKEAHA.120.030620 10.1161/STROKEAHA.120.030620 32933417

[31] Goyal M, McTaggart R, Ospel JM (2022). How can imaging in acute ischemic stroke help us to understand tissue fate in the era of endovascular treatment and cerebroprotection?. Neuroradiology.

[32] Peerlings D, Bennink E, Dankbaar JW (2021). Variation in arterial input function in a large multicenter computed tomography perfusion study. Eur Radiol.

[33] Hsieh J, Nett B, Yu Z (2013). Recent advances in CT image reconstruction. Curr Radiol Rep.

[34] Konstas AA, Goldmakher GV, Lee TY, Lev MH (2009) Theoretic basis and technical implementations of CT perfusion in acute ischemic stroke, Part 2: Technical implementations. AJNR Am J Neuroradiol 30:885–892. 10.3174/ajnr.A1492 10.3174/ajnr.A1492 PMC7051660 19299489

