# Supplementary material for: Standardizing the estimation of ischemic regions can harmonize CT perfusion stroke imaging
Source: Eur Radiol. 2023 Aug 12;34(2):797–807. doi: 10.1007/s00330-023-10035-1 (PMC10853359; doi:10.1007/s00330-023-10035-1)
Supplement: Supplementary file 1 — Supplementary file1 (PDF 902 KB) [file 330_2023_10035_MOESM1_ESM.pdf]

Supplementary material

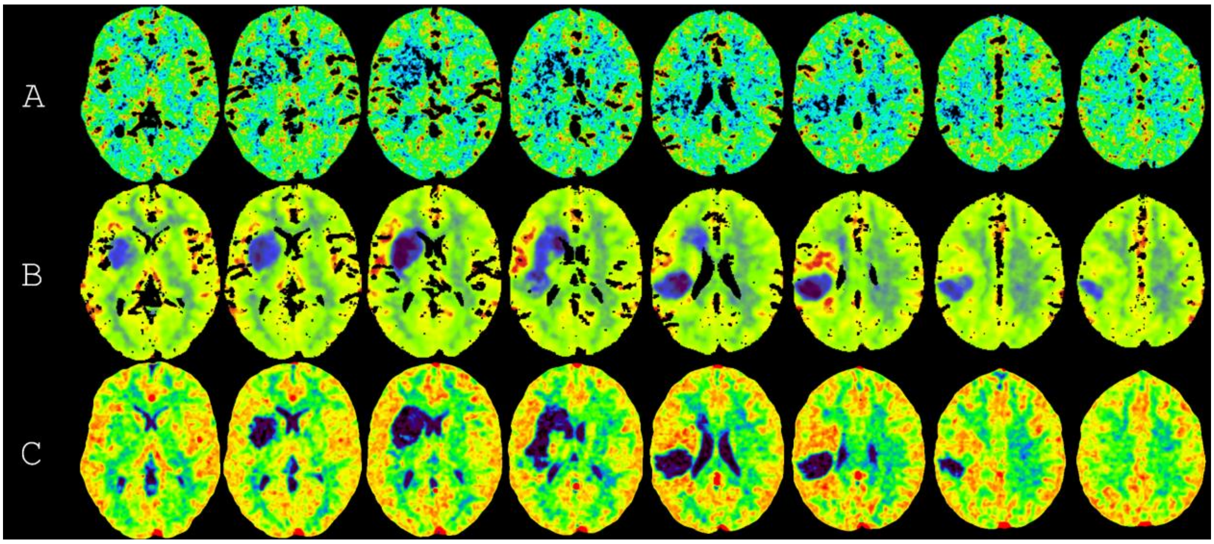

Supplementary Figure 1

*Examples of the cerebral blood volume parameter map generated by the vendor software. In each row, the cerebral blood volume from a different vendor software (A-C) is shown for all slices of the phantom. The color schemes were left unadjusted. The examples are the first noise realization from the representative scan protocols A/4, B/6, and C/1 (see **Error! Reference source not found.**).*

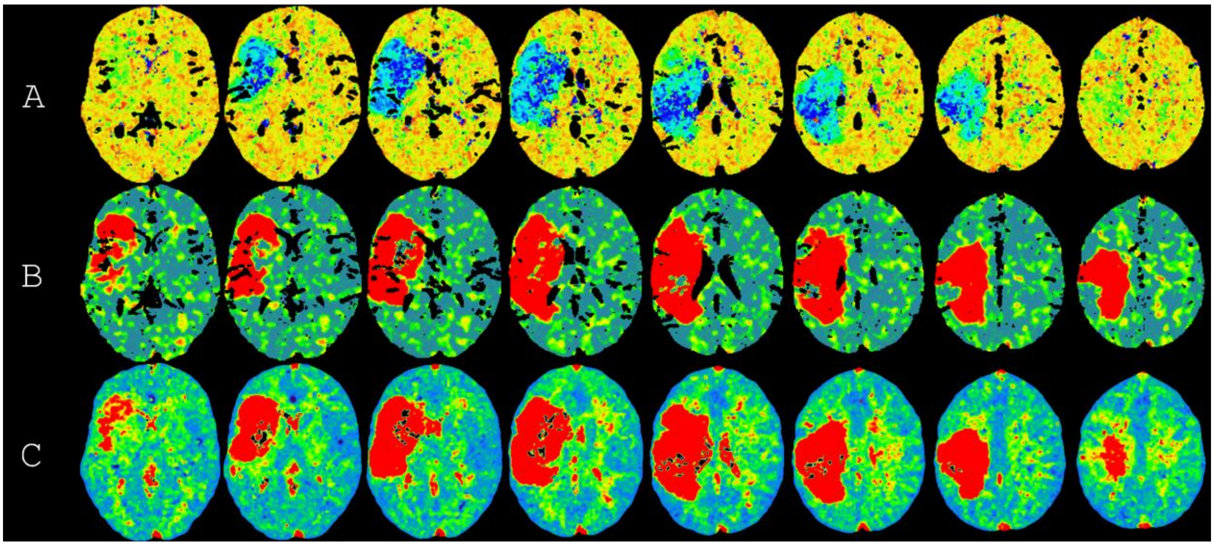

Supplementary Figure 2

*Examples of the mean transit time parameter map generated by the vendor software. In each row, the mean transit time from a different vendor software (A-C) is shown for all slices of the phantom. The color schemes were left unadjusted. The examples are the first noise realization from the representative scan protocols A/4, B/6, and C/1 (see **Error! Reference source not found.**).*

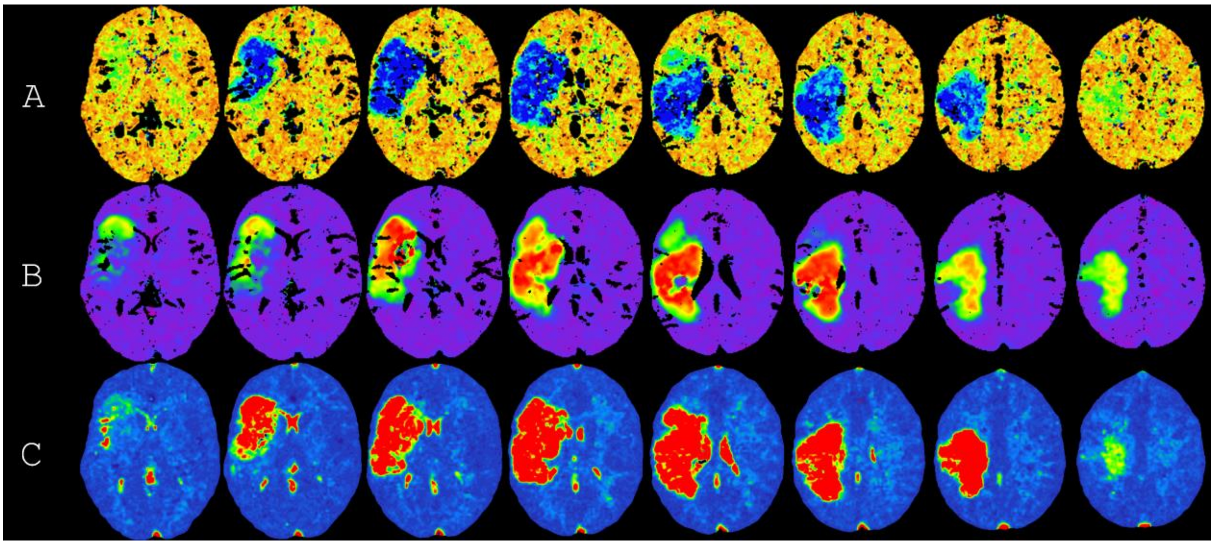

Supplementary Figure 3

Examples of the time to maximum or time to peak parameter map generated by the vendor software. In the first row, the time to peak from vendor software A is shown for all slices of the phantom. In the second and third row, the time to maximum from vendor software B and C are shown for all slices of the phantom. The color schemes were left unadjusted. The examples are the first noise realization from the representative scan protocols A|4, B|6, and C|1 (see ***Error! Reference source not found.****).*

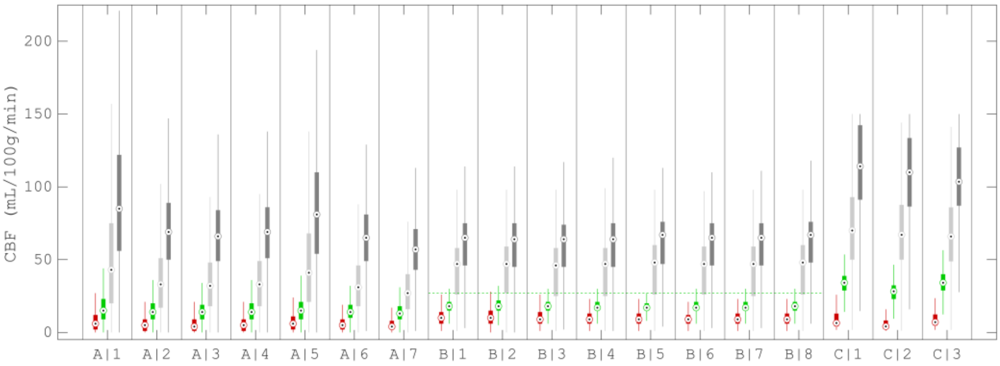

Supplementary Figure 4

Boxplots of the cerebral blood flow (CBF) estimated by the vendor software for each scan protocol (A|1-C|3). We pooled the ten noise realizations of the phantom for each scan protocol. The dashed horizontal colored lines indicate the thresholds given in ***Error! Reference source not found.****, for which relative values were calculated as relative to the median value of the perfusion parameter in healthy matter.*

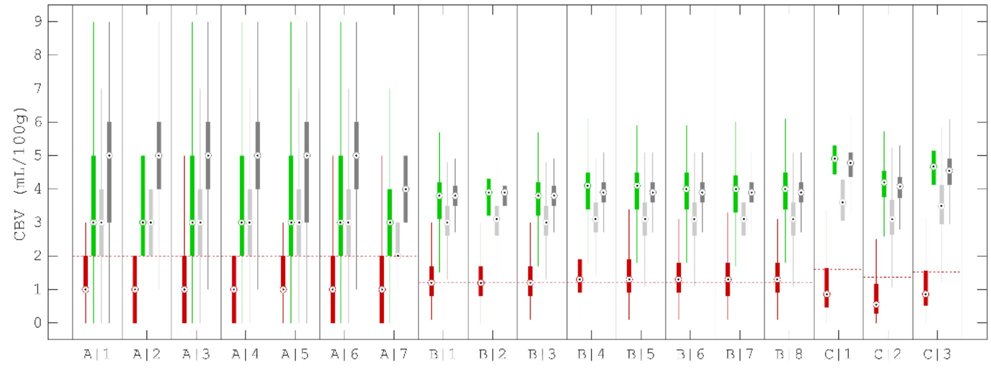

Supplementary Figure 5

Boxplots of the cerebral blood volume (CBV) estimated by the vendor software for each scan protocol (A|1-C|3). We pooled the ten noise realizations of the phantom for each scan protocol. The dashed horizontal colored lines indicate the thresholds given in ***Error! Reference source not found.****, for which relative values were calculated as relative to the median value of the perfusion parameter in healthy matter.*

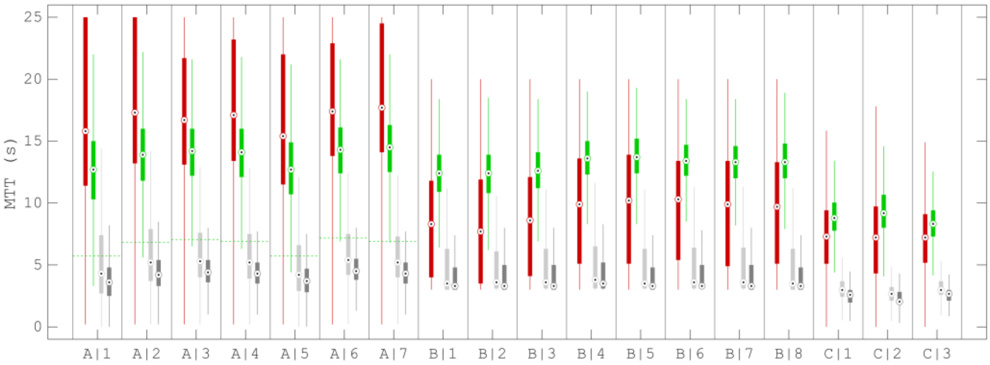

Supplementary Figure 6

Boxplots of the mean transit time (MTT) estimated by the vendor software for each scan protocol (A|1-C|3). We pooled the ten noise realizations of the phantom for each scan protocol. The dashed horizontal colored lines indicate the thresholds given in ***Error! Reference source not found.****, for which relative values were calculated as relative to the median value of the perfusion parameter in healthy matter.*

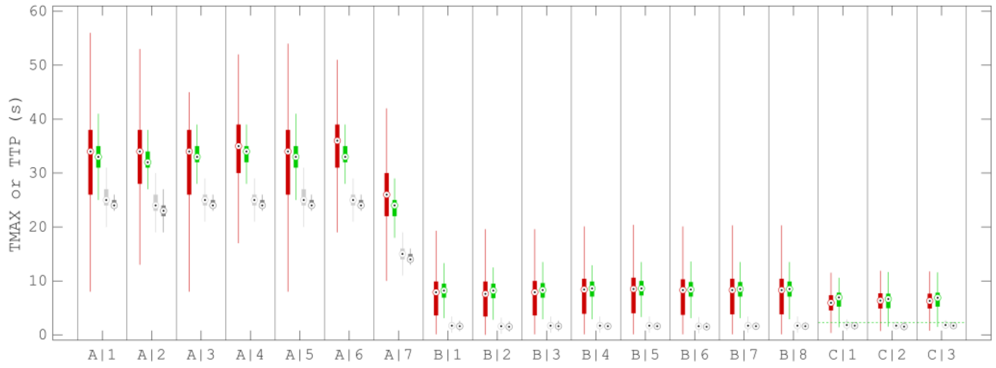

Supplementary Figure 7

Boxplots of the time to maximum (TMAX) or the time to peak (TTP) estimated by the vendor software for each scan protocol (A|1-C|3). For vendor software A, the TTP is shown. For vendor software B and C, the TMAX is shown. We pooled the ten noise realizations of the phantom for each scan protocol. The dashed horizontal colored lines indicate the thresholds given in ***Error! Reference source not found.****, for which relative values were calculated as relative to the median value of the perfusion parameter in healthy matter.*

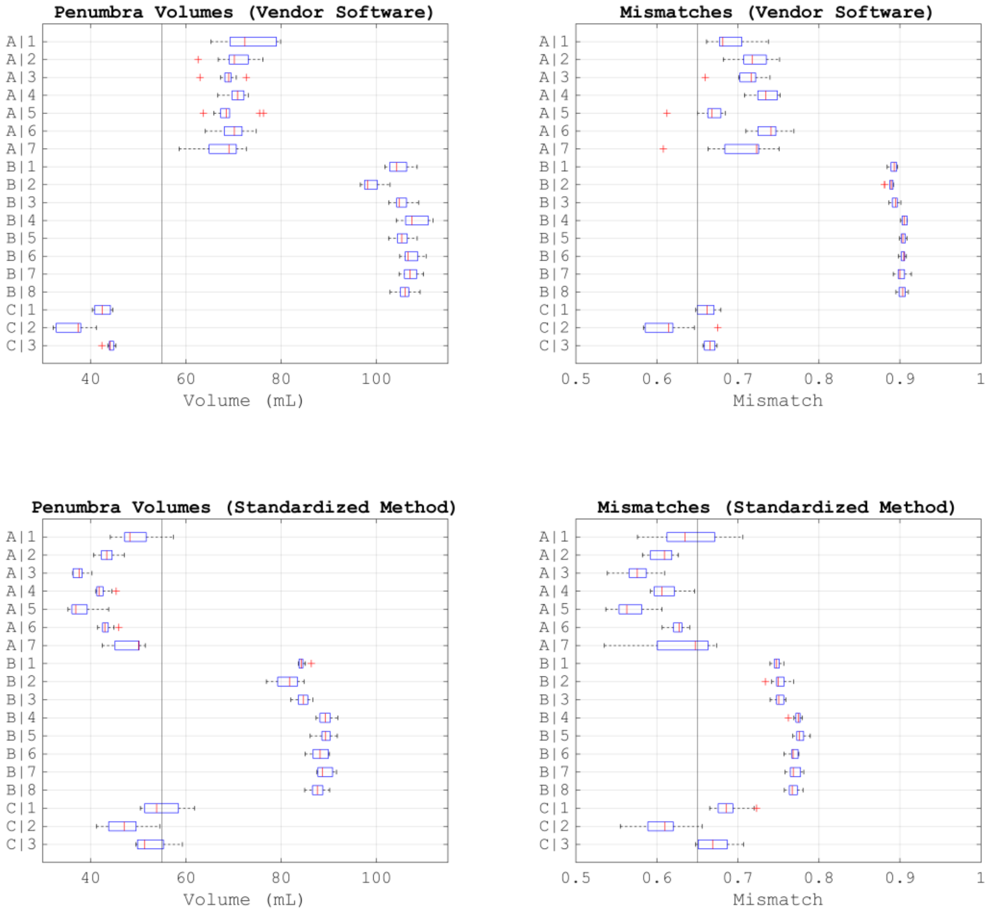

Supplementary Figure 8

Boxplots of the mismatches and the volumes of the penumbra estimated by the vendor software and by the standardized method. The mismatch is defined as the volume of the penumbra over the volume of the hypoperfused tissue. Eighteen scan protocols (A|1-C|3) were analyzed with center-specific software from one of three vendors (A-C). The vertical black lines indicate the ground truth volumes.

Supplementary Table 1

Median [first quartile, third quartile] error of the mismatches and the volumes of the penumbra estimated by the vendor software and by the standardized method. We pooled all noise realizations of the phantom. A positive error indicates overestimation.

**Ischemic region**	**Vendor software**	**Standardized method**
Penumbra (mL)	19.1 [12.5, 50.3]	-1.1 [-11.2, 31.6]
Mismatch (%)	9.8 [3.3, 25.1]	3.9 [-3.5, 11.6]
